# Historical Review about Research on “Bonghan System” in China

**DOI:** 10.1155/2013/636081

**Published:** 2013-06-04

**Authors:** Jun-Ling Liu, Xiang-Hong Jing, Hong Shi, Shu-Ping Chen, Wei He, Wan-Zhu Bai, Bing Zhu

**Affiliations:** Institute of Acupuncture and Moxibustion, China Academy of Chinese Medical Sciences, Beijing 100700, China

## Abstract

The meridian-collateral theory is the theoretical basis of acupuncture-moxibustion therapy. Professor Bonghan Kim, a professor of the Pyongyang Medical University of the Democratic People's Republic of Korea, claimed that he found the anatomical structure of meridian-collaterals, named Bonghan corpuscles (BHCs) and Bonghan ducts (BHDs) system or primo vascular system (PVS), in 1962. From 1963 to 1965, researchers from our institute conducted a series of comparative anatomical experiments, trying to reproduce the so-called BHC- and BHD-like structures in different strains of animals. In the present paper, the authors introduced their research findings about BHC- and BHD-like structures in the young rabbit's umbilicus including its external appearance, ectoplasm and endoplasm, and about strip-like and node-like objects in the blood vessels and lymph vessels near the larger abdominal and cervical blood vessels and chromaffin tissue in the back wall of the rabbit's abdominal cavity and between the bilateral kidneys. In spite of existence of the BHC- and BHD-like structures in the rabbit, there has been no proved evidence for their association with the meridian-collateral system described in acupuncture medicine. In the present historical review, the authors also make a discussion about the significance of those findings.

## 1. Introduction

The theory of meridian-collateral system, established more than two thousand years ago, is the core of acupuncture medicine and the important component of traditional Chinese medicine [1]. In spite of existence of many meridian phenomena in the human body and effective guidance on clinical acupuncture practice, no any corresponding anatomical structure has been found about the main meridian running traces described in many classical works on acupuncture medicine up to now. In 1962, Bonghan Kim, a professor of the Pyongyang Medical University of the Democratic People's Republic of Korea, reported that he observed the electrical responses of acupoints [2]. In 1963, his second report titled “On the acupuncture meridian system” [3] and “On the Kyungrak (meridian) system” [4] were published, claiming that a node-like anatomical structure (Bonghan corpuscles (BHCs), current primo nodes) in the acupoints and a tube-like organ (Bonghan ducts (BHDs), current primo vessels (PVs)) were found on the skin in rabbits. This new organ exists not only in the skin or body surface but also in the internal organs including the blood vessels and lymph vessels. In 1965, his other 3 reports [5–7] were successively published, further explaining the entire network of the Bonghan system (renamed as primo vascular system (PVS)) and the “Sanal” (meaning “live egg,” renamed as Primo-microcell (P-microcell)) which functions in the regeneration and/or repair and hematopoiesis [7]. This so-called greatest discovery (atomic bomb, spacecraft, and PVS) in the 20th century induced a shock to the medical circles of China and other countries in the world. A representative group of Chinese top-ranked medical scientists including anatomist, histologists, physiologists, and pathologists visited the National Acupuncture Meridian Research Institute in Pyongyang twice in 1963. In the meantime, the “Institute of Meridian-Collaterals” which is the main body of the present Institute of Acu-Moxibustion of China Academy of Chinese Medical Sciences was established in Beijing. In addition, several research groups were also founded separately in Beijing, Shanghai, Changchun, and Shenyang for verification of Kim's work [8]. According to Kim's results and animal histological section samples, Chinese medical scientists conducted a series of comparative anatomical studies in the human corpses, rabbits, rats, guinea pigs, cats, dogs, and monkeys, trying to confirm Kim's findings. In Kim's reports and during Chinese scientists' visiting in Pyongyang, no detailed experimental materials, techniques, and histological methods were introduced. Thus, paying great efforts, Chinese medical scientists finally found the similar structure of superficial BHC-like structure in the young rabbit's umbilicus, strip-like and node-like objects in the blood vessels and lymph vessels near the larger abdominal and larger cervical blood vessels, and chromaffin tissue in the back wall of the rabbit's abdominal cavity and between the bilateral kidneys, but failed in reproducing the so-called spontaneous bioelectrical signal propagation of PVs, subcutaneous BHDs or BHCs, and so forth of the acupoint regions in the rabbit's thigh [8]. Due to some special factors at that time, these original results have not been formally published in any domestic or foreign journals until now. Nowadays, some of the Chinese principal top-ranked medical specialists have already passed away and can not see their achievements published. In order to disclose that period of history and to express our respect to their great efforts, we report their results as follows.

## 2. Results

### 2.1. Superficial BHC- and BHD-Like Structures

From the beginning of 1964, Chinese medical scientists employed routine histological perfusion, sectioning, hematoxylin and eosin (H&E) staining, and light microscope and so forth, to conduct a wide variety of experiments in the human corpses, rabbits, rats, guinea pigs, cats, dogs, and monkeys for reproducing superficial BHCs and BHDs in the acupoint regions of the body surface (more than 10 thousands tissue sections have been still reserved in our institute at present), but failed to find the similar structure. At last, they observed BHC- and BHD-like structures in the umbilici of the young rabbits.

#### 2.1.1. Longitudinal Profile of the Rabbit's Umbilicus

**Figure 1:**
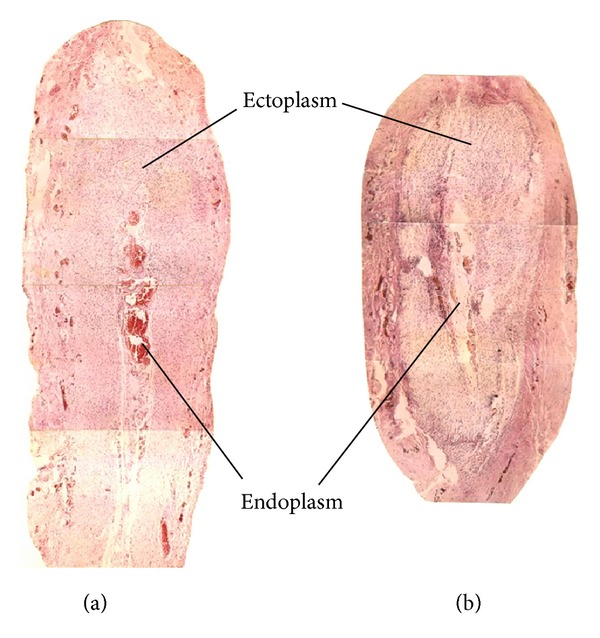
Comparison between the young rabbit's umbilicus observed by Chinese researchers (a) and Bonghan corpuscle (BHC) (b) in the longitudinal section. (a) A photo of hematoxylin and eosin (H&E) staining showing the longitudinal profile of the young rabbit's umbilicus (magnification, ×4), being cord-shaped in outline and about 2 cm in length. (b) A photo of the longitudinal profile of BHC presented by Northern Korean National Acupuncture Meridian Research Institute in 1963.

Following routine transcardiac perfusion, the umbilicus tissue of the anesthetized young rabbit (about a week in age) was removed for observation under light microscope (Leitz, Germany). According to Kim's results and animal tissue section samples provided (Figure 1(b)) during Chinese medical specialists' visiting in 1963, Chinese researchers observed that the young rabbit's umbilicus was near completely the same as superficial BHCs in the external appearance including the shape, size, and arrangement of superficial blood vessels and the longitudinal structure of the umbilicus tissue sections (Figure 1(a)). The rabbit's umbilicus, about 2 cm in length, was a longitudinal cord-like organ with two intumescent endings connecting to the skin on the top and the skeleton muscle near the abdominal cavity at the bottom, respectively (Figure 1(a)). 

**Figure 2:**
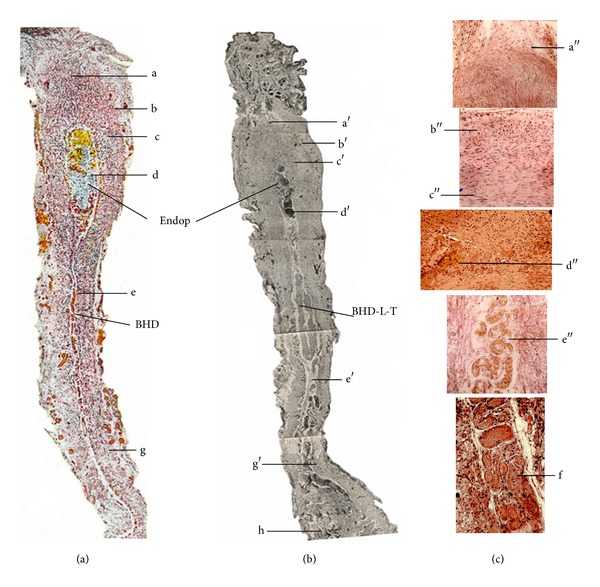
Comparison of histological structure of the superficial BHC (a) and the umbilicus tissue (b), (c) in rabbits. (a) Profile of BHC reported by Professor Kim (*J Jo Sun Med*, vol. 90, 1963 pages 6–35). (b) BHC-like structure of the young rabbit's umbilicus found by Chinese researchers in 1964 (an original photo unpublished) (16 × 6.3). (c) Partial components of the umbilicus (c) stained with H&E method. Both BHC and umbilicus are divided into ectoplasm (composed of smooth muscles, elastic fibers, argyrophilic fibers, etc.) and endoplasm (Endop, containing chromaffin cells, blood vessels, Fibrillenstruktur, etc.) (16 × 16). BHD: Bonghan duct, BHD-L-T: BHD-like tubule; a, a′, a′′: radiation-like smooth muscle; b, b′, b′′: outer-circular layer smooth muscle; c, c′, c′′: entolongitudinal layer smooth muscle; d, d′,  d′′: chromaffin cells; e, e′, e′′: blood vessels, f: Fibrillenstruktur, g, g′: elastic fibers, and h: skeleton muscle.

By using a freezing microtome (Leitz, Germany), the umbilicus tissue was successively sectioned (40 *μ*m) and observed using a light microscope. According to Professor Kim, the BHC was mainly composed of ectoplasm and endoplasm. Under microscope, the rabbit's umbilicus tissue on the longitudinal section was also composed of ectoplasm and endoplasm (Figures 2(a) and 2(b)). 

#### 2.1.2. Ectoplasm of the Umbilicus

From the top to the bottom of the longitudinal umbilicus tissue, the ectoplasm contained skin layer, smooth muscles, and elastic fibrous tissue (Figure 2(b)). The smooth muscles included radiation-like smooth muscle fibers extending toward the skin (Figure 2(b), a′, a′′), thinner outer-circular layer (Figure 2(b)  b′, b′′), thicker entolongitudinal layer between the skin and the endoplasm (Figure 2(b) c, c′, c′′), and fewer muscle fibers near the skeleton muscle of the abdominal cavity. Apparently, the components of the umbilicus were basically identical to those of BHC-BHD complex. The running direction of the smooth muscle was along the longitudinal axis of the umbilicus. 

These smooth muscles, encompassed by a thin layer of collagen fibers or elastic fibers, were mainly distributed in the epidermis layer near the skin of the umbilicus. The elastic fibers gradually appeared and increased in the amount on the lower end of the ectoplasm. Warthin-Starry dyeing displayed that the ectoplasm of the umbilicus also contained richer argyrophilic fibers. Feulgen staining showed that in the epidermis layer of the umbilicus, there existed some hemisphere-like cell clusters. 

#### 2.1.3. Endoplasm of the Rabbit's Umbilicus

Under light microscope, some chromaffin cells (Figure 2(c), d′′), crooked blood vessels (Figure 2(c), e′′), Fibrillenstruktur (Figure 2(c), f′′), follicles, and smooth-muscle-like cells were observed in the endoplasm of the umbilicus. The Fibrillenstruktur was stained to be either blue (basophilia) or red (acidophilia) with H&E method. In addition, there also existed a type of cell (similar to chromaffin cell) containing a smaller nucleus and rich cytoplasma with yellow-brown granules. Further analysis showed that this type of cell is phagocyte swallowing red blood cells and so forth, and the yellow-brown granules are hemofuscin. In the neighboring part of the cavity of the follicle-like structure, some basophilia granules and smooth muscle-like cells were observed. 

### 2.2. Bonghan Duct-Like Object in the Deep Tissues of the Rabbit

**Figure 3:**
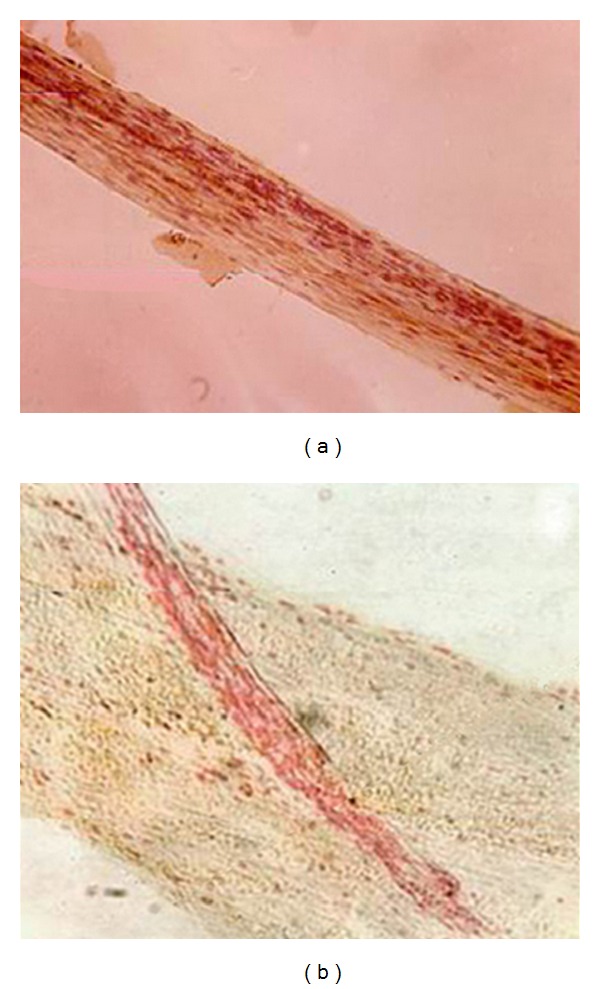
Photos of intravascular strip-like object (a) in the rabbit's blood vessels found by Chinese researchers and extravascular BHD (b) in the rabbit's deep tissue reported by Professor Kim, being similar in the outline. (a) A photo of H&E staining showing the strip-shaped object in the rabbit's blood vessels (16 × 16). (b) Deep BHD attached to a blood vessel reported by Professr Kim.

After trying various perfusion methods, Chinese researches ultimately found strip-shaped (Figure 3(a)) and node-like objects in the larger blood vessels and lymph vessels in the rabbit. Feulgen dyeing showed that these objects contained rhabdocyte nucleus and chromonucleic acid granules. After H&E staining, these intravascular rhabdocyte nuclei and chromonucleic acid granules were found to be deformed leukocyte nuclei or deciduous vascular endotheliocyte nuclei and had no chromonucleic acid granules. Following perfusion with normal saline mixed with anticoagulation reagent (heparin), these strip-shaped and node-like objects in the blood vessels disappeared. In the intravenous node-like objects, some divided hematopoietic cells were found. 

### 2.3. Bonghan Corpuscle-Like Object in the Deep Tissues of the Rabbit

**Figure 4:**
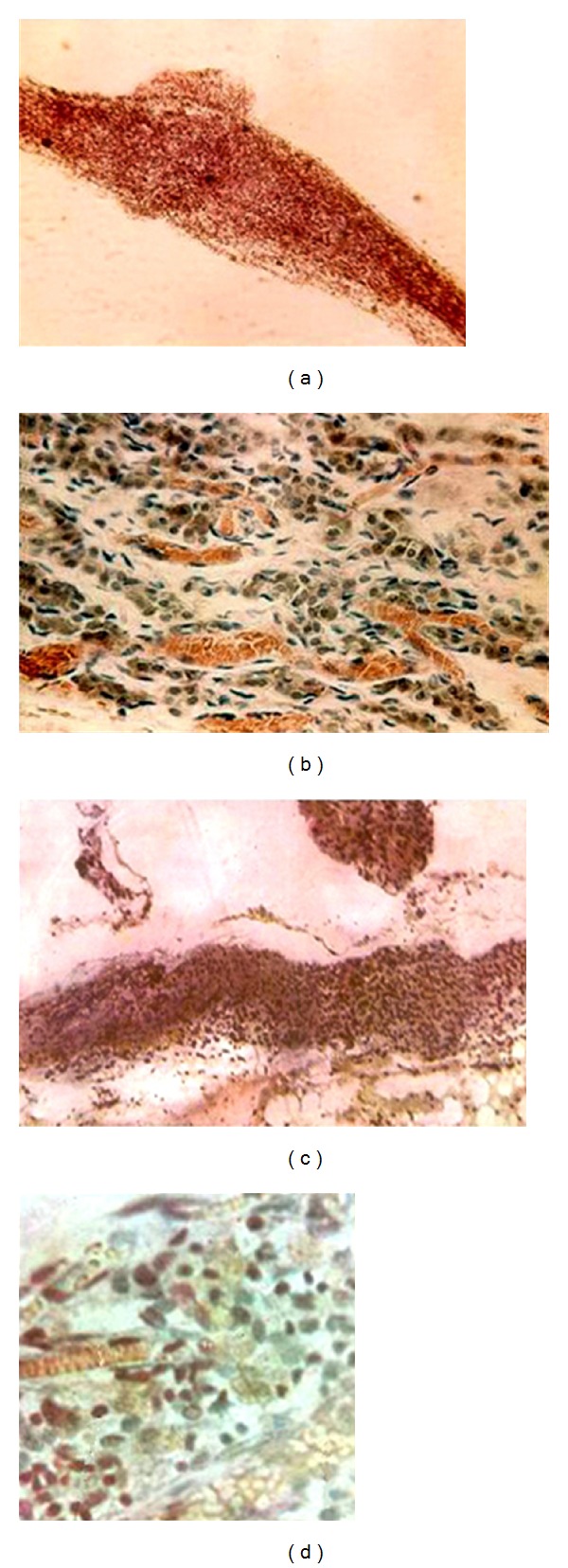
Comparison of the rabbit's node-like object in the blood vessels and lymph vessels (a) and chromaffin tissue (c) in the back wall of abdominal cavity found by Chinese researchers and deep BHC (b) and chromaffin tissue (d) reported by Professor Kim. (a, c; magnification: 16 × 10).

In the neighboring tissues of the larger abdominal vessels and cervical blood vessels, Chinese researchers found some node-like objects which were connected to ducts at their two ends and had no smooth muscles in the rabbit (Figure 4(a)). Following sectioning and staining, these node-like objects and their connected ducts were found to be lymph nodes and lymph vessels, respectively. 

In the back wall of the rabbit's abdominal cavity and the tissues between the two kidneys, Chinese researchers observed some chromaffin cells (Figure 4(c)). Histological analysis confirmed the observed tissue to be chromaffin tissue near the paraganglion. These node-like objects and chromaffin tissue were similar to deep BHCs in the outline (Figures 4(b) and 4(d)).

## 3. Discussion

Just as those mentioned in the preface of the present paper, Chinese researchers tried various methods to reproduce superficial BHCs and BHDs in different strains of animals and human corpses and finally observed BHC-like structure in the young rabbit's umbilicus and deep BHD-like structure in the abdominal vessels and lymph node-like object (BHC) around the rabbit's cervical vessels. But in the young rabbit's umbilicus, no transverse BHD-like tubules along the subcutaneous tissue of the body and no rich chromonucleic acid in the BHD-like structures were found, being different to Kim's results [5]. The superficial BHC- and BHD-like structure of the rabbit's umbilicus were also not reproduced in the umbilicus of the human body and other animals. In addition, the superficial BHC- and BHD-like objects were not found in the acupoint region of the rabbit's thigh where Professor Kim claimed [3]. According to Professor Kim's reports on the schematic structure of BHCs [3, 5], the young rabbit's umbilicus was also composed of ectoplasm and endoplasm connecting to a BHD-like tubule extending toward the abdominal cavity. 

The components of the ectoplasm in the umbilicus including the location or distribution and arrangement of the smooth muscles (near the skin and fibrous tissue near the abdominal muscle) and radiation-like smooth muscle fibers, the outer circular and entolongitudinal layers, thickness of the smooth muscles, the thin layer of collagen fibers or elastic fibers encompassing the smooth muscles, the distribution, shape, and size of the argyrophilic fibers, and the hemisphere-like cell clusters in the umbilicus are near completely identical to BHCs found in the acupoint and skin by Kim [3, 5]. 

In the endoplasm of the superficial BHCs, there was a follicle-like structure formed by several layers of cubic endothelial cells which were also observed in the young rabbit's umbilicus. The other components of the endoplasm such as the chromaffin cells, Fibrillenstruktur, follicles, smooth-muscle-like cells and crooked blood vessels, and the phagocytes containing yellow-brown granules are also the same as those of BHCs [3, 5]. In fact, the endoplasm originates from the residual collagen fibers of the degenerated blood vessels in the umbilicus. 

The external appearance of the umbilicus is also very similar to that of BHC sample (provided by Kyungrak Institute of Korea in 1963 during Chinese medical scientists' visiting in Pyongyang, unpublished results of research work briefings from our institute in 1965) in the shape and blood vessel distribution. Thus, the aforementioned young rabbit's umbilicus is quite similar to BHC in the shape and structure. 

Moreover, the strip- (thread-) shaped and node-like objects in the abdominal vessels and the lymph node-like objects around the larger cervical blood vessels are also consistent with those of BHDs and BHCs in the deep tissues of the rabbit [3, 5]. And the thread-like structure is also the same as the results reported by Soh and colleagues [9–11]. 

In spite of a similar outline between the intravascular node-like or thread-like objects and intravascular BHCs and BHDs, Chinese researchers from our institute thought that these objects were no more than some blood clots at that time. Because after perfusion with anticoagulation reagent, the node-shaped objects disappeared. This is identical to the result of Chinese Changchun research group [8] who also observed a white thread-shaped object in the rabbits' thoracic aorta (extending to the abdominal aorta) following femoral vein perfusion with normal saline. If the perfusion fluid was mixed with adrenaline which facilitates blood coagulation, the thread-shaped objects could be seen not only in the main stems of the thoracic aorta and abdominal aorta but also in their branches. If the perfusion fluid was mixed with heparin (resisting blood coagulation), the thread-shaped objects disappeared completely. Thus, Chinese researchers held that these intravascular thread-like objects were probably a fibrin bundle related to blood clotting [8]. This conclusion is not insistent to Soh and colleagues' report that the blood fibrin and PV are two different objects distinguishable by using Acridine orange staining and fluorescent microscope [12]. 

The deep BHC-like objects containing chromaffin cells and lymphocyte-like cells found in the rabbit's abdominal cavity and the tissues between the two kidneys were identical to BHCs reported by Kim [4]. The chromaffin cells exist mainly in the adrenal medulla, sympathetic ganglia, and so forth and function in secreting adrenaline, noradrenaline, and so forth in the human body. Whether these chromaffin cells are involved in functional activities of the meridian-collateral system or not, there have been no any reports up to now. 

In 1960s, Professor Kim's work induced great attention not only in China but also in some countries of the world. Fujiwara (an assistant professor in anatomy) and Yu from Osaka City University Medical School found the superficial BHC-like structure in one rabbit and observed many BHCs inside the blood vessels and on the surface of the internal organs in the rabbit [13]. In the contemporaneity, Kellner, a histologist from Austria [14], conducted a series of animal experiments and confirmed the existence of the extremity corpuscle-like objects reported by Kim, but failed to find the structure proclaimed by Kim. He thought that these objects were merely a residual structure of embryonic development period and were impossible to have any physiological functions. 

It is very clear that all the aforementioned structures in the rabbit are known tissues or appeared under some special conditions. However, in the human body, the umbilicus is just the site of Shenque (CV8) acupoint of the conception vessel which functions in regulating borborygmus, diarrhea, edema, chronic enteritis, prolapse of the rectum, and various deficiency syndromes [15]. However, clinical effects of acupuncture or moxibustion stimulation on gastrointestinal activities and other organs rely on the integrity of the nerve system, neurotransmitters, body fluid, and gastrointestinal hormones [16]. Last year, Wang and her colleagues [17] observed that neither electrical stimulation nor section of the PVs on the surface of the stomach or intestine could affect gastric motility, and ST36-electroacupuncture (EA) stimulation induced increase of gastric systolic and diastolic activities in rats. It suggests that, at least, the thread-like structure on the surface of the gastrointestinal tract has no definite association with the effects of EA stimulation. 

Furthermore, after surveying the available publications from China and other countries on the question “what is being stimulated in acupuncture?”, Chan [18] held that the most convincing results indicate that expression of acupuncture effects definitely involves the nervous system. On the extremity of the body, acupuncture is equated to direct nerve stimulation. Acupuncture stimulation initiated sensory signal input from the acupoint to the brain and then the resulting processing, integration, and balancing regulation of the physiological and pathological information in the central nervous system are the basis for producing clinical effects [19, 20]. For instance, the abolition of acupuncture analgesia by injection of local anesthetics into acupoints is probably the strongest evidence to support the conclusion that neural innervation is required for the acupuncture stimulation response [21, 22]. Longhurst [23] pointed out that although additional research is warranted to investigate the role of some of the structures (such as tendinomuscular structures, primo vessels, higher- or lower-temperature traces, low skin resistance, etc.) identified, it seems clear that the peripheral and central nervous system can now be considered to be the most rational basis for defining meridians. 

In the recent 10 years, analysis of PVS has been resurrected by Soh and his colleagues from the Department of Physics in the Seoul National University [24, 25] and Ma et al. from China [26]. They used Trypan blue dyeing and other techniques to show that PVs exist not only on the surface of the internal organs, but also in the nerve system, cardiovascular system, lymphatic system, fascia of the abdominal cavity, adipose tissue, generative system (testis), skin and abdominal wall, primo fluid and microcells, egg vitelline membrane, and cancer [27] in rabbits, rats, mice, dogs, and pigs. They have observed the structure of PVs by using modern instruments as electron microscope and laparoscope [28, 29] and analyzed their protein profiles of PVs tissue using proteomic technique [30], claiming that their results will shift the level of the oriental medicine from the traditional wisdom and art with a long history to the biomedical sciences. Recently, Kwon and colleagues [11, 31] observed microscopic nodes and ducts inside lymphatics, as well as on the surface of internal organs in the rat. The nodes contain a variety of immune cells, usually enriched with mast cells, eosinophils, neutrophils and histiocytes, as well as chromaffin cells, other granule-containing cells. They guess that these ducts may be involved in the transport of secretory granules from the nodes and serve a new circulatory system.

Generally speaking, the anatomical structure always accompanies functional roles in the human body and other animals. But the conjecture about the connection between PVS and meridian-collateral system does not have any proved experimental basis. We do hope to get more proved and universally accepted evidence, particularly on its physiological functions under both normal and pathological conditions. 

The meridian-collateral system theory is founded more than two thousand years ago. It highlights the close association among different parts of the body surface, and between the body surface and internal organs, functioning in regulating physiological activities of various systems of the human body via neuron-endocrine-immune networks. Despite objective existence of many meridian phenomena in the human body [32–35] and limited explanations by using current biological theories, any acupuncture intervention outcomes deviating from predominate roles of the neuron-endocrine-immune network will not be accepted by the academic circle, and the conjecture is probably unreasonable. 

## 4. Conclusion

Chinese researchers found superficial BHC-like objects only in the young rabbits' umbilici, and also observed deep BHC- and BHD-like objects in the abdominal and cervical blood vessels. But they could not distinguish the deep BHC- and BHD-like objects from coagulation. No BHC- and BHD-like structures were found in other animals and human corpses as well as in the acupoint regions. No any proved evidence for the association between the PVS or Bonghan system and meridian-collateral system of acupuncture medicine has been found up to now.
